# Supplementary material for: Nuclear membrane protein SUN2 promotes replication of flaviviruses through modulating cytoskeleton reorganization mediated by NS1
Source: Nat Commun. 2024 Jan 5;15:296. doi: 10.1038/s41467-023-44580-6 (PMC10766649; doi:10.1038/s41467-023-44580-6)
Supplement: Supplementary file 1 — Supplementary information [file 41467_2023_44580_MOESM1_ESM.pdf]

Supplementary data

Nuclear membrane protein SUN2 promotes replication of flaviviruses through modulating cytoskeleton reorganization mediated by NS1

Yanxia Huang[1,2,3#], Qinyu Peng[1,2#], Xu Tian[1,2#], Cancan Chen[4], Xuanfeng Zhu[1,2], Changbai Huang[1,2], Zhiting Huo[1,2], Yang Liu[5], Chao Yang[3,6*], Chao Liu[1,2*], Ping Zhang[1,2*]

[1]Key Laboratory of Tropical Diseases Control (Sun Yat-sen University), Ministry of Education, Guangzhou, China
[2]Department of Immunology and Microbiology, Zhongshan School of Medicine, Sun Yat-sen University, Guangzhou, China
[3]Department of Neurosurgery, First Affiliated Hospital of Sun Yat-sen University, Guangzhou, 510080, China
[4]Department of Pathology, First Affiliated Hospital of Sun Yat-sen University, Guangzhou, 510080, China
[5]School of Biomedical Sciences, Li Ka Shing Faculty of Medicine, The University of Hong Kong, Hong Kong SAR, China
[6]Department of Neurosurgery, Guangxi Hospital Division of The First Affiliated Hospital, Sun Yat-sen University

* Correspondence:
zhangp36@mail.sysu.edu.cn (PZ); liuchao9@mail.sysu.edu.cn (CL); ychao@mail.sysu.edu.cn (CY)

This PDF contains Supplementary figures, figure legends, and tables.

Supplementary Figures and figure legends

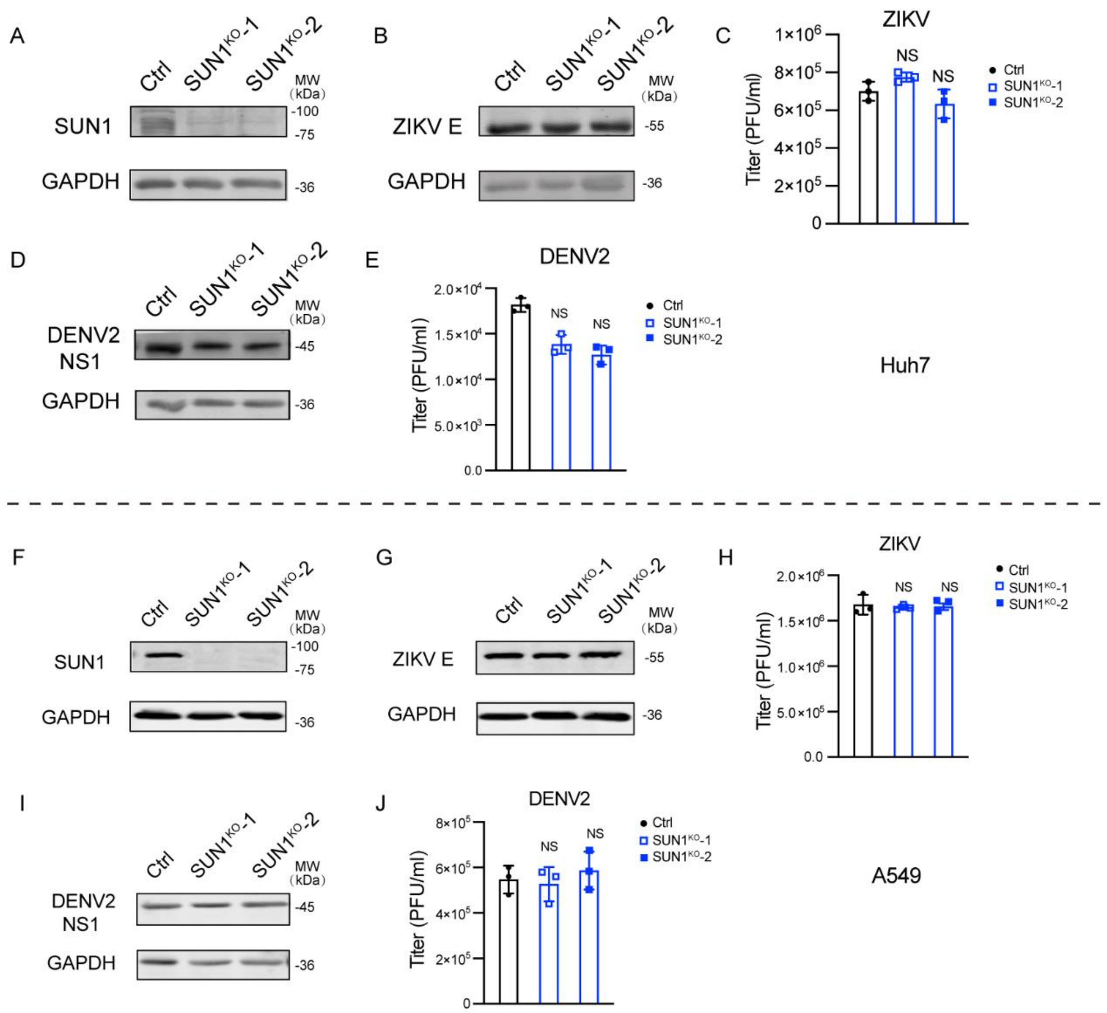

Supplementary Figure 1. SUN1 is dispensable for ZIKV and DENV2 infection. (A-E) Impact of SUN1 knockout on ZIKV or DENV2 replication in Huh7 cells. (A) Knockout efficiency of two SUN1 knockout clones (SUN1^{KO}-1 and SUN1^{KO}-2) in Huh7 cells were confirmed by western blot using anti-SUN1 antibody. GAPDH was probed as a loading control. (B-E) Control and SUN1^{KO} cells were infected with ZIKV (B-C) or DENV2 (D-E) at an MOI of 3. At 24 h p.i., cells were harvested for western blot (B and D) and supernatants were collected for plaque assay (C and E). (F-H) Impact of SUN1 knockout on ZIKV or DENV2 replication in A549 cells. Two SUN1 knockout clones (SUN1^{KO}-1 and SUN1^{KO}-2) in A549 cells were validated by western blot (F). (G-J) Cells were infected with ZIKV (G-H) or DENV2 (I-J) at an MOI of 3. At 24 h p.i., cells were harvested for western blot (G and I) and supernatants were collected for plaque assay (H and J). (A, B, D, F, G, and I) Representative images of three independent experiments are shown. (C, E, H, and J) The data are shown as mean ± SD, n=3 biologically independent experiments. Statistical significance was determined using two-tailed unpaired *t* test (NS, no statistical significance). Source data are provided as a Source Data file.

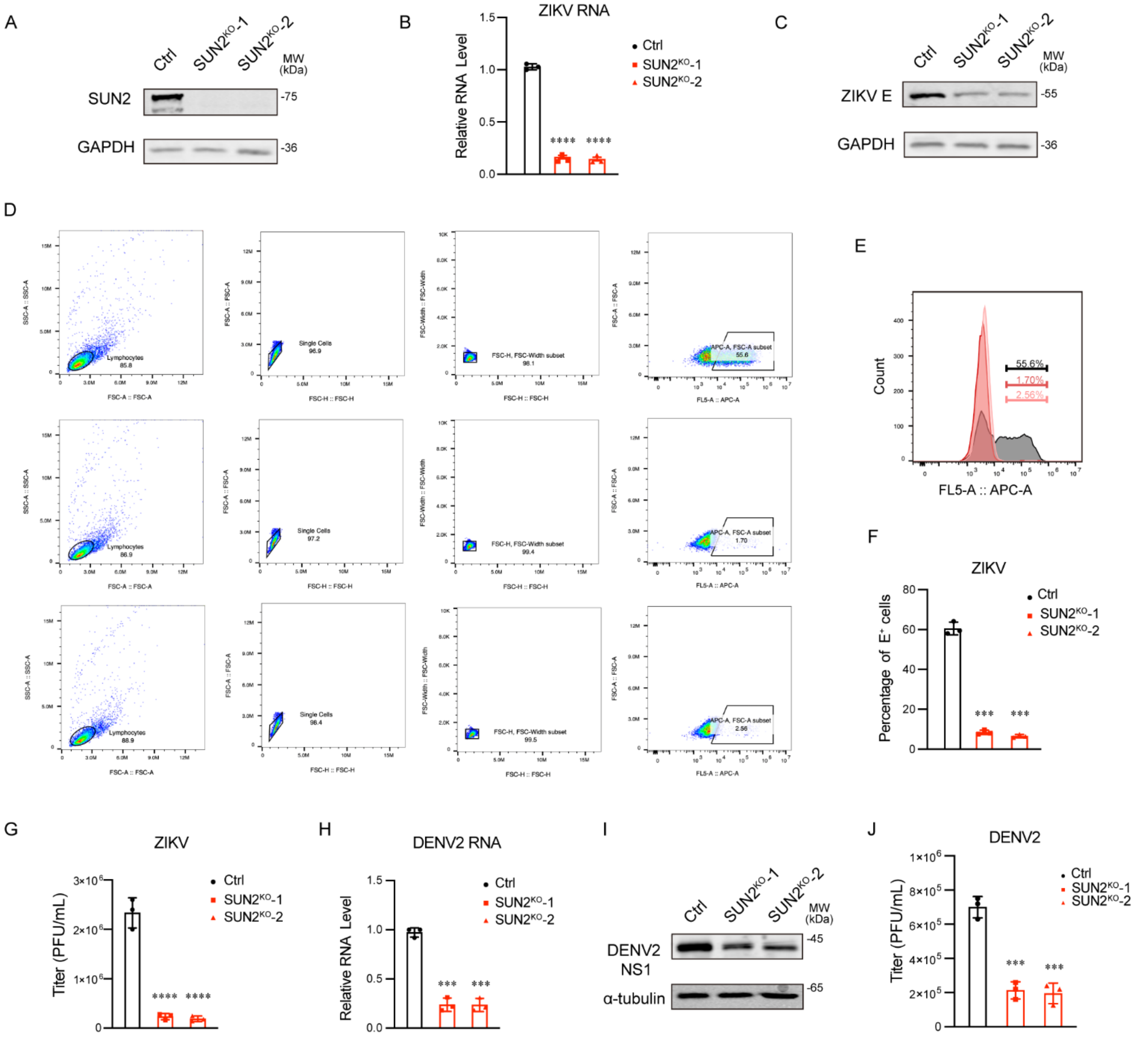

Supplementary Figure 2. SUN2 promotes flavivirus infection in A549 cells. (A) Knockout efficacy of two SUN2 knockout clones ($SUN2^{KO}$-1 and $SUN2^{KO}$-2) in A549 cells were confirmed by western blot using anti-SUN2 antibody. GAPDH was probed as a loading control. (B-C), Control and $SUN2^{KO}$-1 and $SUN2^{KO}$-2 cells were infected with ZIKV at an MOI of 3. At 24 h p.i., cells were harvested for qRT-PCR (B), western blot (C). (D-F), Control and $SUN2^{KO}$-1 and $SUN2^{KO}$-2 cells were mock- infected or infected with ZIKV at an MOI of 3. At 24 h p.i., cells were harvested by staining with ZIKV E followed by flow cytometry. (D) Sequential gating strategy used to analyze infected cells (ZIKV E^+) for Supplementary Fig. 2E and 2F. (G) Control and $SUN2^{KO}$-1 and $SUN2^{KO}$-2 cells were infected with ZIKV at an MOI of 3. At 24 h p.i., supernatants were collected for plaque assay. (H-J) Control and $SUN2^{KO}$ A549 cells were infected with DENV2

at an MOI of 3. At 24 h p.i., cells were harvested for qRT-PCR (H), or western blot (I), and supernatants were collected for plaque assay (J). (A, C, I) Representative images of three independent experiments are shown. (B, F, G, and J) The data are shown as mean ± SD, n=3 biologically independent experiments. Statistical significance was determined using two-tailed unpaired *t* test (**, P < 0.01; ***, P < 0.001; ****, P < 0.0001). *p* values: (B) $p < 0.0001$(SUN2^{KO}-1), $p < 0.0001$ (SUN2^{KO}-2); (F), $p = 0.0006$ (SUN2^{KO}-1), $p = 0.0007$ (SUN2^{KO}-2); (G) $p < 0.0001$(SUN2^{KO}-1), $p < 0.0001$ (SUN2^{KO}-2); (H), $p = 0.0001$ (SUN2^{KO}-1), $p = 0.0001$ (SUN2^{KO}-2); (J), $p = 0.0001$ (SUN2^{KO}-1), $p < 0.0001$ (SUN2^{KO}-2). Source data are provided as a Source Data file.

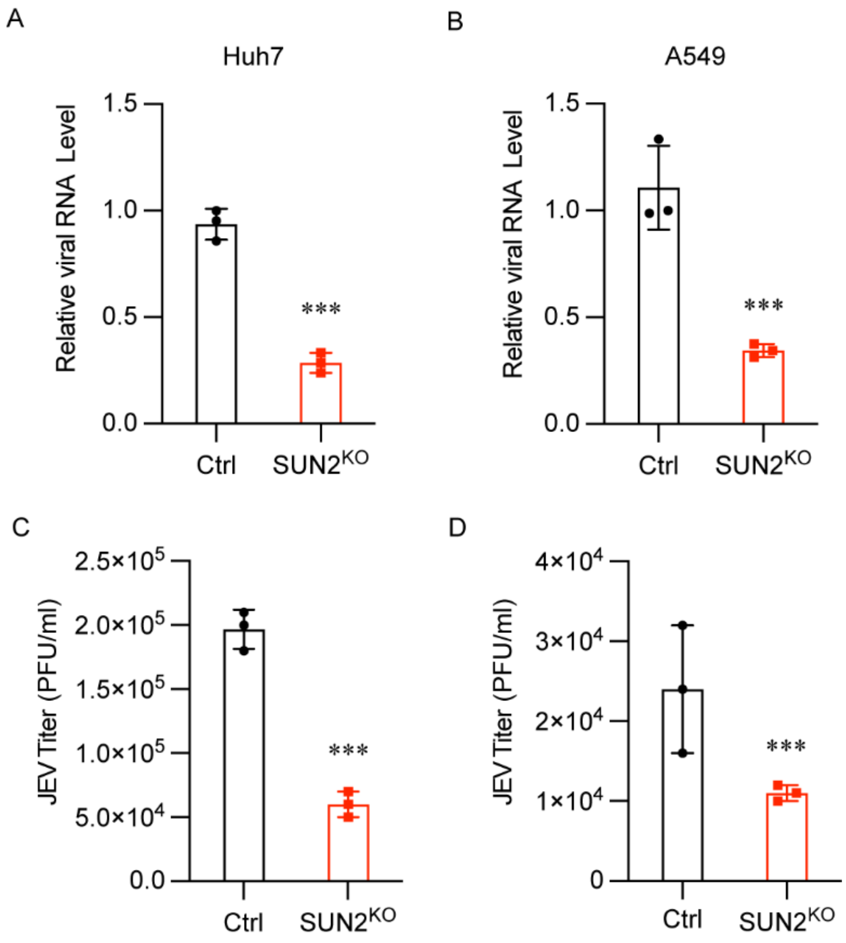

Supplementary Figure 3. SUN2 promotes JEV infection in Huh7 and A549 cells. Control and SUN2^{KO} Huh7 and A549 cells were infected with JEV at an MOI of 3. Cells were harvested at 24 h p.i. for qRT-PCR (A and B), and supernatants were collected for plaque assay (C and D). The data are shown as mean ± SD, n=3 biologically independent experiments. Statistical significance was determined using two-tailed unpaired *t* test (***, $P < 0.001$). *p* values: (A) $p = 0.0002$, (B) $p = 0.0006$, (C) $p = 0.0002$, (D) $p = 0.0004$. Source data are provided as a Source Data file.

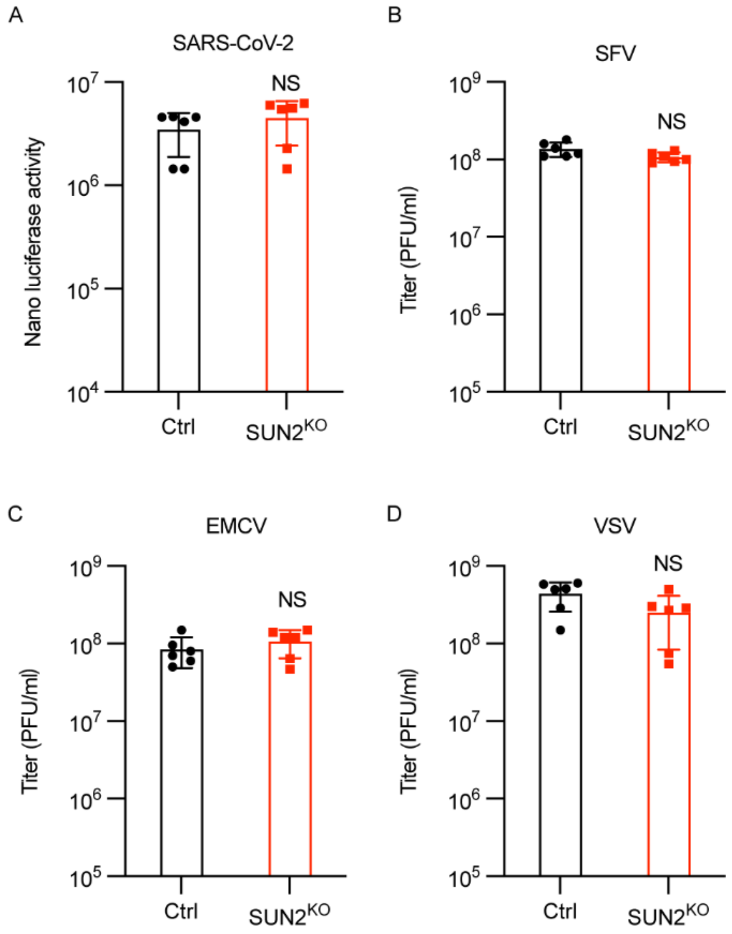

Supplementary Figure 4. SUN2 is dispensable for non-flavivirus infection.
(A) SARS-CoV-2 replicon assay. Control and SUN2^{KO} Huh7 cells were transfected with SARS-CoV-2 replicon. Cells were harvested at 24 h p.i. for the luciferase assay. (B-D) Control and SUN2^{KO} Huh7 cells were infected with SFV (B), EMCV (C), or VSV (D) at an MOI of 3. At 24 h p.i., cells and supernatants were collected for plaque assay. The data are shown as mean ± SD, n=6 biologically independent samples. Statistical significance was determined using two-tailed unpaired *t* test (NS, no statistical significance). Source data are provided as a Source Data file.

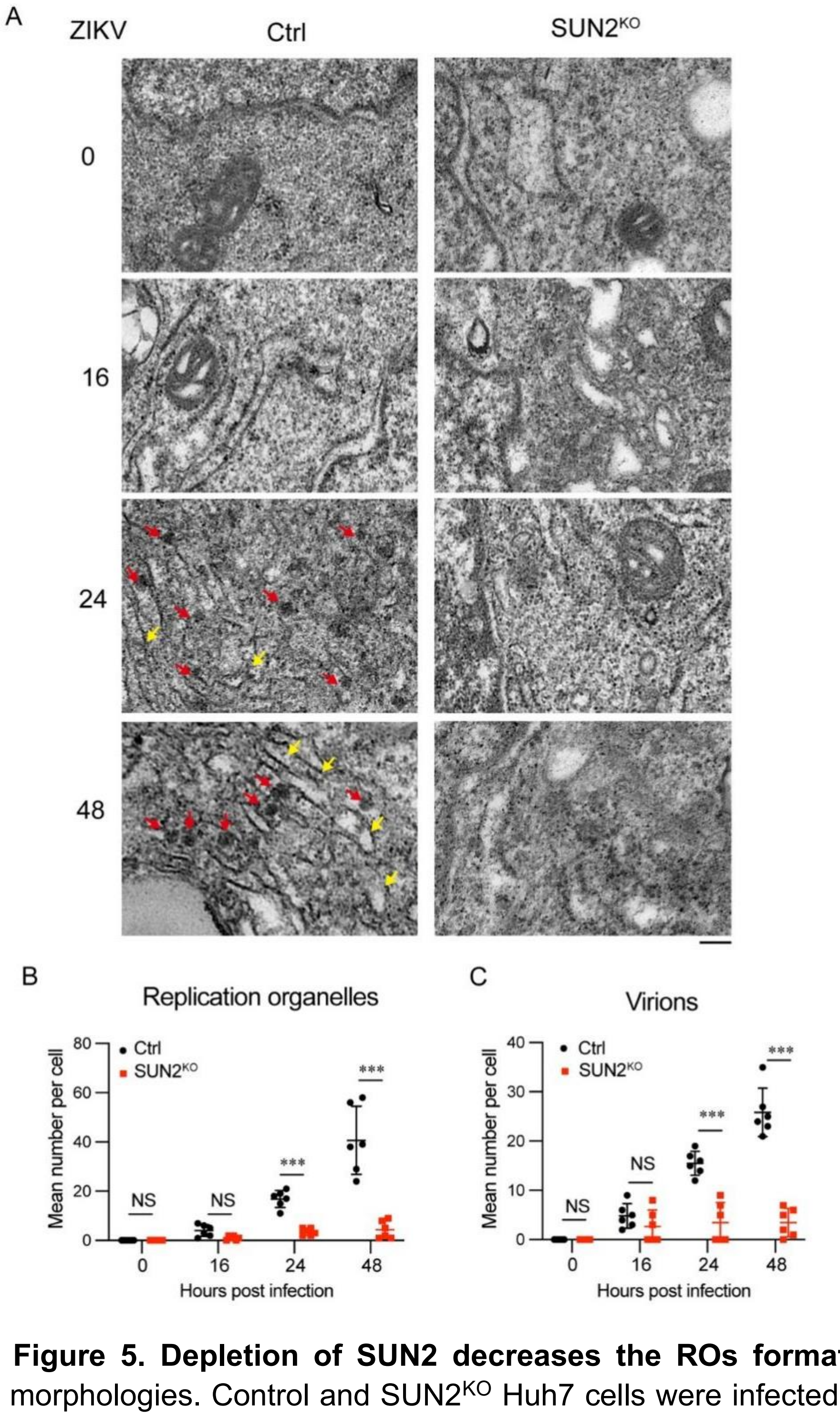

Supplementary Figure 5. Depletion of SUN2 decreases the ROs formation and virion amount. (A) ER morphologies. Control and $SUN2^{KO}$ Huh7 cells were infected with ZIKV at an MOI of 3. Cells were fixed and processed for TEM analysis at 0, 16, 24, and 48 h p.i. ROs were indicated with yellow arrows and viral particles were indicated with red arrows. Representative images of three independent experiments are shown. Scale bars, 100 nm. (B-C) Quantitation of ROs (B), and virions (C) in control and $SUN2^{KO}$ cells (n = 6 cells per condition). Graphs show

the mean number per cell counted; error bars represent Mean ± SD. Statistical significance was determined using two-way ANOVA (NS, no statistical significance; ***, $P < 0.001$). p values: (B) $p = 0.0004$ (24 h), $p = 0.0005$ (48 h), (C) $p = 0.0001$ (24 h), $p = 0.0004$ (48 h). Source data are provided as a Source Data file.

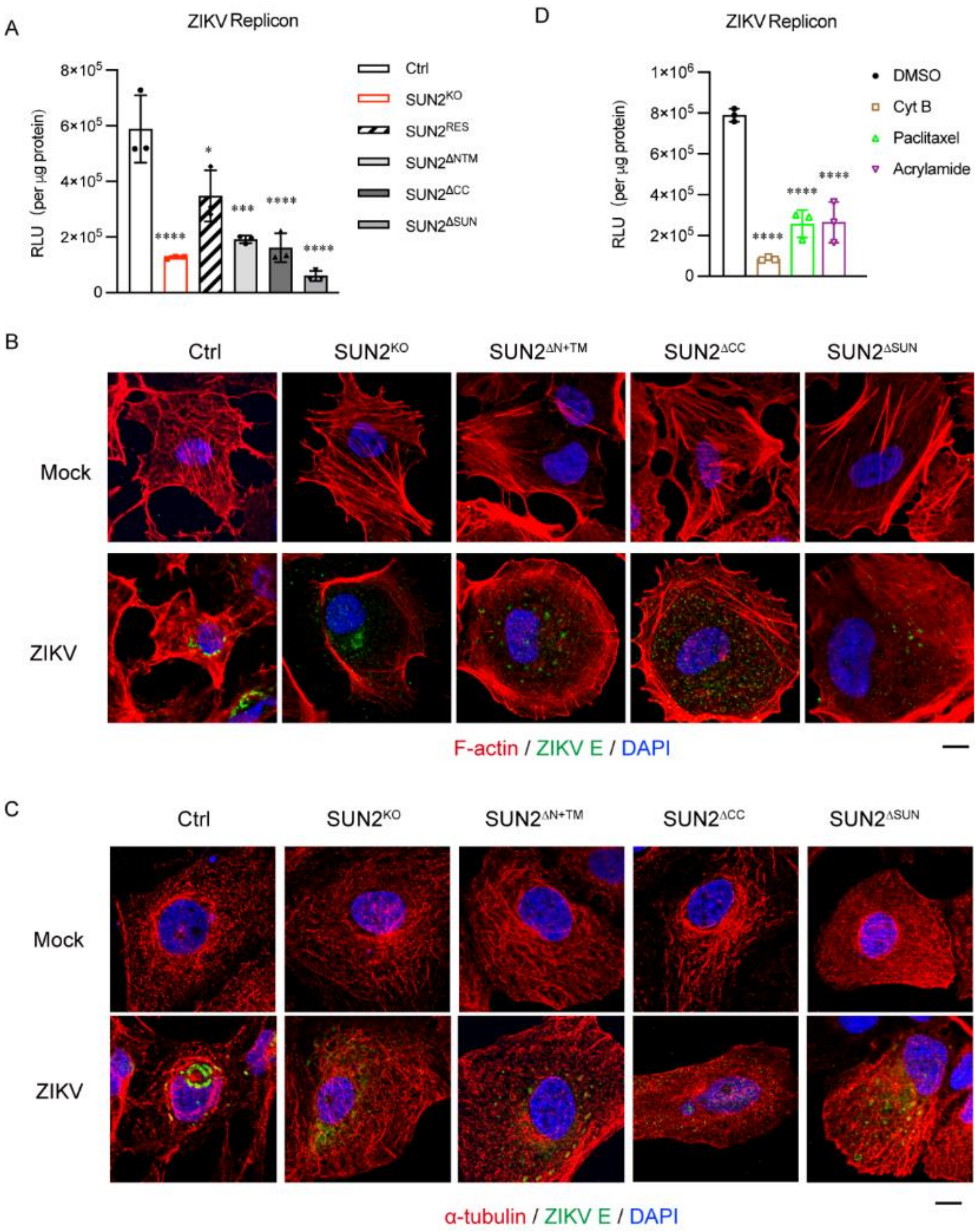

Supplementary Figure 6. Three regions of SUN2 are essential for rearrangement of cytoskeleton induced by ZIKV. (A) Replicon assay. Control, SUN2^{KO}, SUN2^{RES}, SUN2$^{\Delta N+TM}$,

SUN2$^{\Delta CC}$, and SUN2$^{\Delta SUN}$ cells were transfected with ZIKV WT replicon RNAs. Cells were harvested at 24 h p.t. for luciferase assay. The data are shown as mean ± SD, n=3 biologically independent experiments. Statistical significance was determined using two-way ANOVA (*, P < 0.1; ***, P < 0.001; ****, P < 0.0001). *p* values: $p < 0.0001$ (SUN2^{KO}), $p = 0.0510$ (SUN2^{RES}), $p = 0.0001$ (7 SUN2$^{\Delta N+TM}$), $p < 0.0001$ (SUN2$^{\Delta CC}$), $p < 0.0001$ (SUN2$^{\Delta SUN}$). (B, C) IFM assay. Control, SUN2^{KO}, SUN2$^{\Delta N+TM}$, SUN2$^{\Delta CC}$, and SUN2$^{\Delta SUN}$ cells were infected with ZIKV at an MOI of 8. At 24 h p.i., cells were fixed and stained to visualize ZIKV envelope (green), and nucleus (blue), F-actin (B, red), and α-tubulin (C, red). Representative images of three independent experiments are shown. Scale bars, 10 μm. Representative images of at least three independent experiments are shown. (D) Replicon assay. Huh7 cells were treated with cytochalasin B (10 μM), paclitaxel (10 μM), or acrylamide (2 μM) for 1 hour. Huh7 cells were transfected with ZIKV WT replicon RNAs, and harvested at 24 h p.t. for luciferase assay. The data are shown as mean ± SD, n=3 biologically independent experiments. Statistical significance was determined using two-way ANOVA (****, P < 0.0001). *p* values: $p < 0.0001$ (Cyt B, paclitaxel, or acrylamide). Source data are provided as a Source Data file.

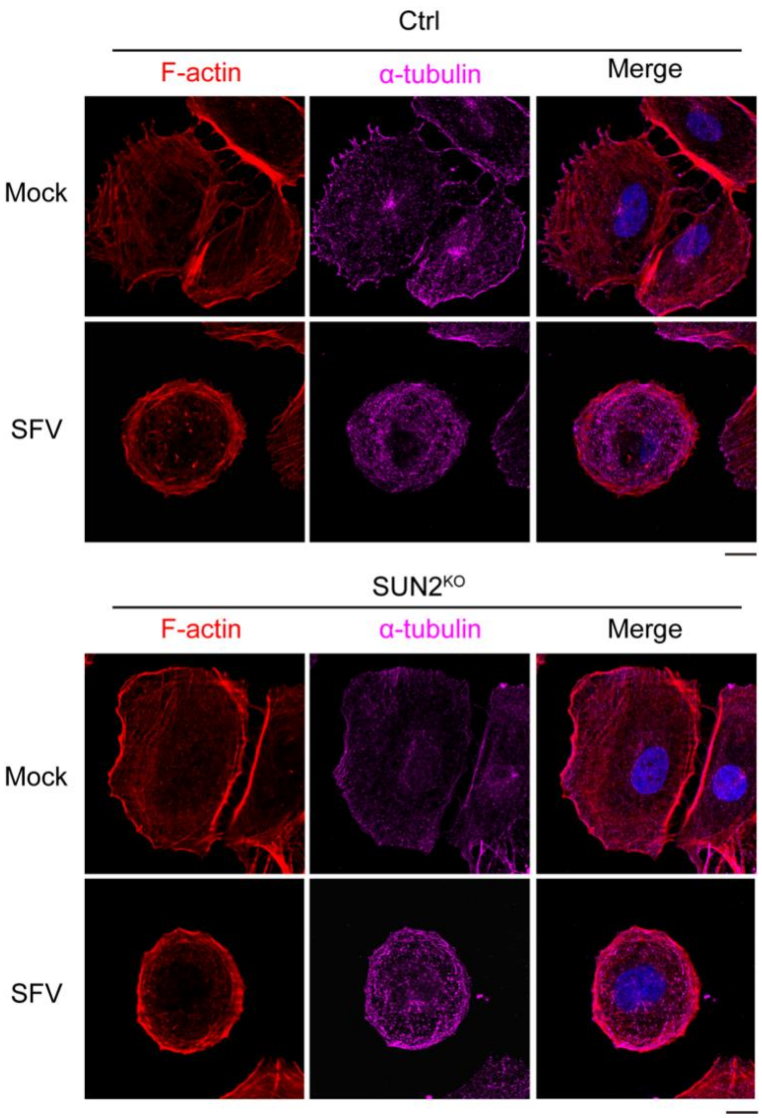

Supplementary Figure 7. SUN2 does not affect rearrangement of F-actin and α-tubulin induced by SFV infection. Control and SUN2^{KO} Huh7 cells were infected with SFV at an MOI of 3. Cells were fixed at 9 h p.i. and stained to visualize F-actin (red), α-tubulin (magenta) and nucleus (blue). Representative images of three independent experiments are shown. Scale bars, 10 μm.

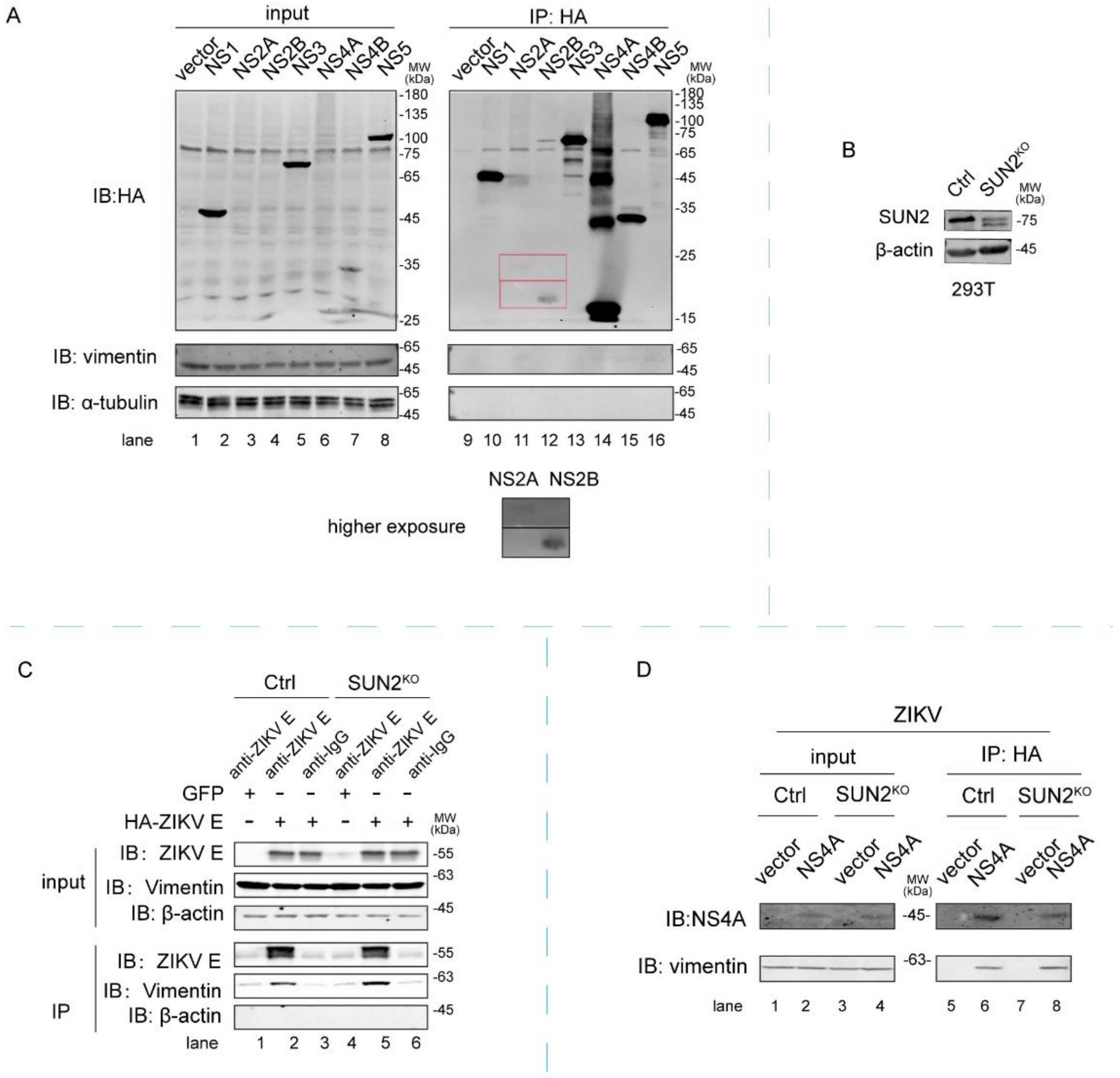

Supplementary Figure 8. Interaction between ZIKV proteins and cytoskeleton proteins. (A) 293T cells were transfected with plasmids expressing ZIKV HA-NS proteins for 48 h. Whole-cell extracts were prepared for co-IP assay using anti-HA agarose beads. Samples were detected by western blot using anti-HA, anti-vimentin, and anti-α-tubulin antibodies. (B) Western blot to confirm the knockout efficiency of SUN2 knockout 293T cells. (C) Control and $SUN2^{KO}$ 293T cells were transfected with the plasmid expressing ZIKV E protein for 48 h. Whole-cell extracts were prepared for co-IP assay using anti-E antibody. Samples were detected by western blot using anti-E, anti-vimentin, and anti-β-actin antibodies. (D) Control and $SUN2^{KO}$ Huh7 cells were transfected with plasmid expressing HA-NS4A, followed by infection with ZIKV at an MOI of 5 at 12 h p.t. Whole-cell extracts were harvested at 24 h p.i. for co-IP assay using anti-HA agarose beads. Samples were detected by western blot using anti-NS4A and anti-vimentin antibodies. Representative images of three independent experiments are shown. Source data are provided as a Source Data file.

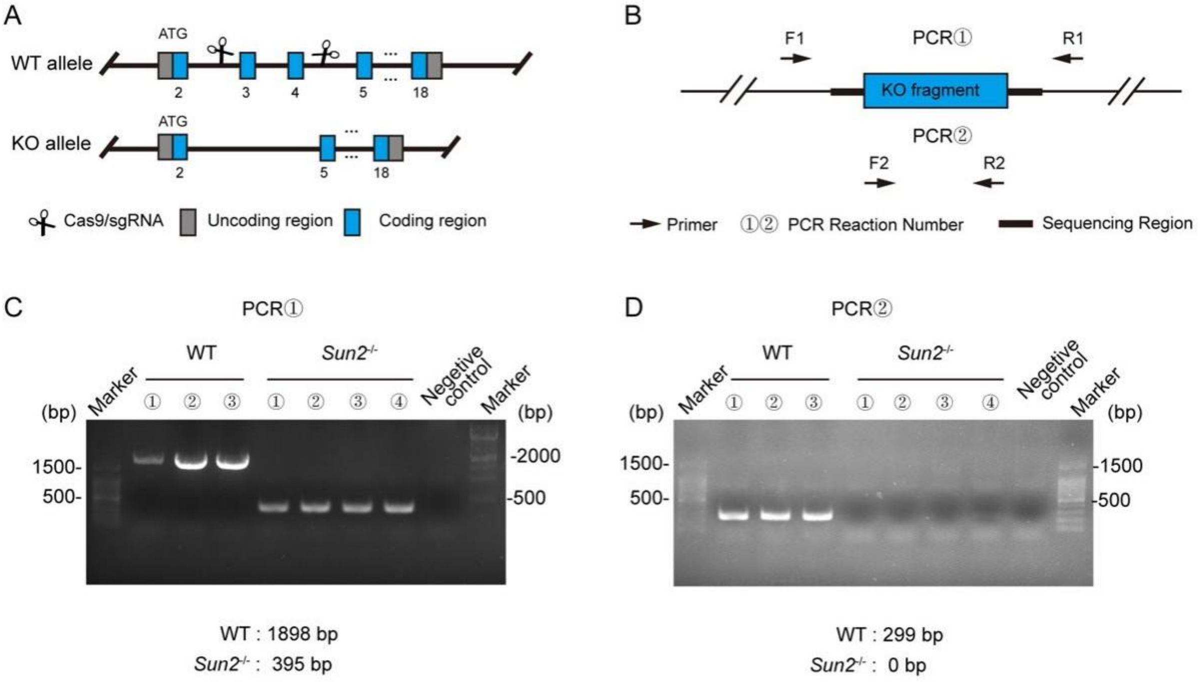

Supplementary Figure 9. CRISPR/Cas9-based *Sun2* knockout strategy and verification of knockout effect in mice. (A) Schematic diagram showed the CRISPR/Cas9 technology editing *Sun2* gene. (B) Schematic diagram showed the positions of PCR primers for genotyping. (C, D) Genotyping of *Sun2* in mice by PCR. The PCR products of WT mice are 1898 (primer set 1) or 299 (primer set 2) bp, and *Sun2* knockout *mice are* 395 (primer set 1) or 0 (primer set 2) bp. Representative images of three independent experiments are shown. Source data are provided as a Source Data file.

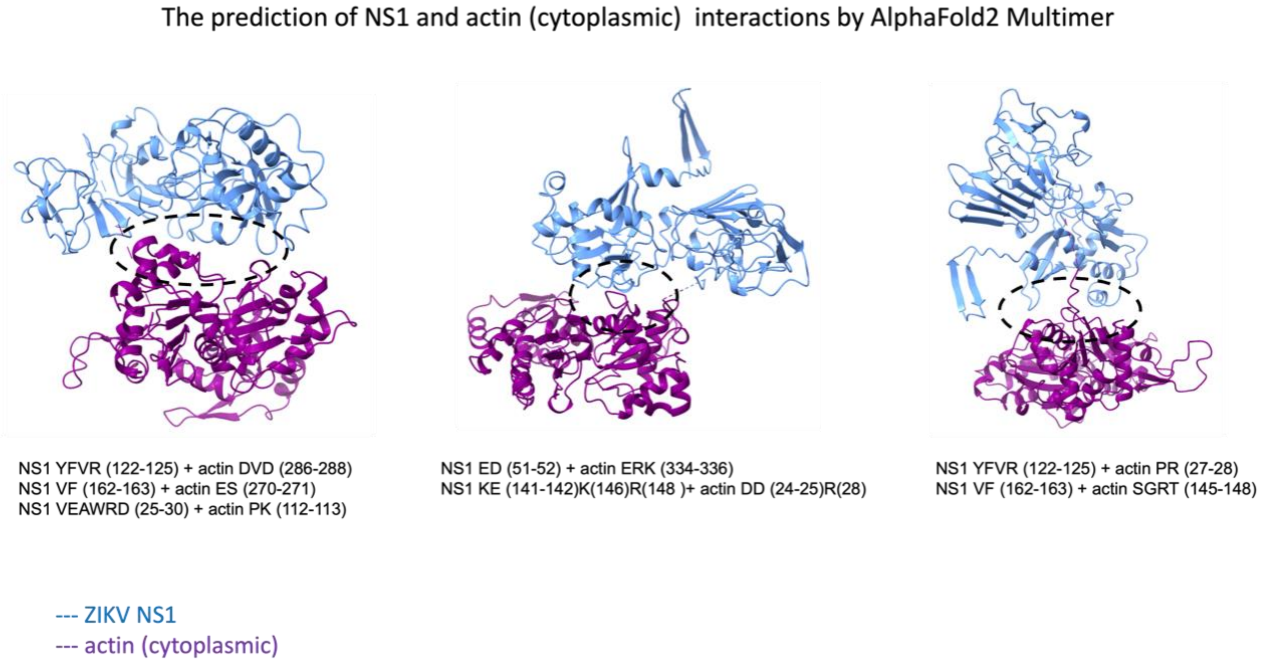

Supplementary Figure 10. Prediction of potential interactions between NS1 and actin by AlphaFold2. Structural prediction of ZIKV NS1 and host actin (cytoplasmic), and their potential interaction modes by AlphaFold2. The amino acids involved in the interaction interfaces are indicated under the predicted models.

Supplementary Tables

Supplementary Table 1. Primary antibodies used in western blot, IFM, and IHC

Antibody	Source	Identifier
SUN1 rabbit polyclonal Ab	Proteintech	24568-1-AP
SUN2 rabbit polyclonal Ab	Proteintech	27556-1-AP
ZIKV E rabbit polyclonal Ab	GeneTex	GTX133314
ZIKV E mouse monoclonal Ab	GeneTex	GTX634155
ZIKV NS1 rabbit polyclonal Ab	GeneTex	GTX133307
ZIKV NS3 rabbit polyclonal Ab	GeneTex	GTX133320
ZIKV NS4A rabbit polyclonal Ab	GeneTex	GTX133704
ZIKV NS5 rabbit polyclonal Ab	GeneTex	GTX133312
DENV2 NS1 mouse monoclonal Ab	National Cheng Kung University	N/A
HA-tag monoclonal Ab	MBL	M180-3
β-actin mouse monoclonal Ab	Sigma	A1928
vimentin mouse monoclonal Ab	Proteintech	60330-1-Ig
α-tubulin mouse monoclonal Ab	Ray Antibody Biotech	RM2007
Calnexin rabbit polyclonal Ab	Proteintech	10427-2-AP
Nesprin-1 rabbit monoclonal Ab	Abcam	Ab192234
Nesprin-1 rabbit monoclonal Ab	HUABIO	ET7107-28
mCherry rabbit polyclonal Ab	Proteintech	26765-1-AP
GAPDH rabbit monoclonal Ab	Proteintech	10494-1-AP

Supplementary Table 2. Sequences of primers used in qRT-PCR

Gene	Sequence (5’-3’)
ZIKV *NS1*	5F, GTCAGAGCAGCAAAGACAA<br>3R, CAGCCTCCTTTCCCTTAACA
DENV2 *C*	5F, TCCTAACAATCCCACCAACAGCA<br>3R, AGTTCTGCGTCTCCTGTTCAAGA
β-actin	5F, GCTCCTCCTGAGCGCAAG<br>3R, CATCTGCTGGAAGGTGGACA

Supplementary Table 3. Sequences of primers used in CRISPR/Cas9 editing

Gene	Primer	Sequence (5’-3’)
SUN1	sgRNA1-5F<br>sgRNA1-3R	CGGGCTACACGTATGCGCTC<br>GAGCGCATACGTGTAGCCCG
SUN1	sgRNA2-5F<br>sgRNA2-3R	GTCCCGCCGTAGTTTGCGCC<br>GGCGCAAACTACGGCGGGAC
SUN2	sgRNA1-5F<br>sgRNA1-3R	CAGCAGCGGAGGGAGCTCGG<br>CCGAGCTCCCTCCGCTGCTG
SUN2	sgRNA2-5F<br>sgRNA2-3R	CGCCTCACGCGCTACTCCCA<br>TGGGAGTAGCGCGTGAGGCG

Supplementary Table 4. Sequences of oligonucleotides used in cloning

Primers	Sequence (5'-3')
SUN2-CDS	5F, ATGTCCCGAAGAAGCCAGCGC<br>3R, CATGGGGAGCCCGCCCACTAG
SUN2-RES-sgRNA1	5F, AGCAGCGGAGGGAGTTCAGTAGCAGGAGCTGGGAGTCAG<br>3R, CTGACTCCCAGCTCCTGCTACTGAACTCCCTCCGCTGCT
ΔN+TM	5F, ATGAAGGACAGCAGGAGGCCG<br>3R, CATGGGGAGCCCGCCCACTAG
ΔCC-1	5F, ATGTCCCGAAGAAGCCAGCGC<br>3R, CAGCGTCAGGCTCAGAACACGCTGCTCAGC
ΔCC-2	5F, GCTGAGCAGCGTGTTCTGAGCCTGACGCTG<br>3R, CATGGGGAGCCCGCCCACTAG
ΔSUN	5F, ATGTCCCGAAGAAGCCAGCGC<br>3R, TACGCCCTGGAGTCAGGAGGGTAG
E	5F, ATCAAACAAGTTTGTACAAAAAAGC<br>3R, ATCGAACCACTTTGTACAAGAAAGC
NS1	5F, ATGGATGTGGGGTGCTC<br>3R, TGCAGTCACCATTGACCTTAC
NS2A	5F, ATGGGATCAACTGATCACATG<br>3R, CCGCTTCCCACTCCTTGTGA
NS2B	5F, ATGAGCTGGCCCCCTA<br>3R, CCTTTTTCCAGTCTTCACGTA
NS3	5F, ATGAGTGGTGCTCTATGGGAT<br>3R, TCTTTTCCCAGCGGCAAACTC
NS4A	5F, ATGGGAGCGGCTTTTGGAG<br>3R, GGCGGTAATCAAGCCCAGAAG
NS4B	5F, ATGAATGAACTCGGATGGTTGGA<br>3R, ACGTCTCTTGACCAAGCCAGC
NS5	5F, ATGGGGGGTGGAACAGG<br>3R, CAGCACTCCAGGTGTGGACCC
mCherry	5F,<br>TTCCATTTCAGGTGTCGTGAGGATCCATGGTGAGCAAGGGCGAGGA<br>3R,<br>CGGCCGCCCTCGAGGAATTCACGCGTCTTGTACAGCTCGTCCATGC
mCherry-SEC61B	5F,<br>GCATGGACGAGCTGTACAAGGGTGGTGGTCCTGGTCCGACCCCCAGT<br>GG<br>3R,<br>CTAGAGCGGCCGCCCTCGAGGAATTCCTACGAACGAGTGTACTTGC
DN-KASH1	5F,<br>GCATGGACGAGCTGTACAAGGGTGGTGGTCGCGGCTTCCTGTTCAGA<br>GT<br>3R,<br>CTAGAGCGGCCGCCCTCGAGGAATTCTCAGAGTGGAGGAGGGCCAT

DN-KASH2	5F, GCATGGACGAGCTGTACAAGGGTGGTGGTGCAGTGAGAACTACAGAAGG<br>3R, CTAGAGCGGCCGCCCTCGAGGAATTCCTATGTGGGGGGTGGCCCAT

Supplementary Table 5. Sequences of primers used in SUN2^{KO} mice validation

Primers	Sequence (5’-3’)
PCR①	5F, ACGACAGAGTTGTCGTAAGCTGG<br>3R, AATAGCACTGACTGCTTCGAGC
PCR②	5F, CTGTCTCTAACTGACGGGTTTGC<br>3R, TCGAACCACAGACTCGCTGTAGTAG
