# Supplementary material for: Nuclear membrane protein SUN2 promotes replication of flaviviruses through modulating cytoskeleton reorganization mediated by NS1
Source: Nat Commun. 2024 Jan 5;15:296. doi: 10.1038/s41467-023-44580-6 (PMC10766649; doi:10.1038/s41467-023-44580-6)
Supplement: Supplementary file 3 — Reporting Summary [file 41467_2023_44580_MOESM3_ESM.pdf]

nature portfolio

Corresponding author(s): Chao Yang, Chao Liu, Ping Zhang

Last updated by author(s): Dec 4, 2023

Reporting Summary

Nature Portfolio wishes to improve the reproducibility of the work that we publish. This form provides structure for consistency and transparency in reporting. For further information on Nature Portfolio policies, see our Editorial Policies and the Editorial Policy Checklist.

Statistics

For all statistical analyses, confirm that the following items are present in the figure legend, table legend, main text, or Methods section.

n/a	Confirmed	
☐	☒	The exact sample size (*n*) for each experimental group/condition, given as a discrete number and unit of measurement
☐	☒	A statement on whether measurements were taken from distinct samples or whether the same sample was measured repeatedly
☐	☒	The statistical test(s) used AND whether they are one- or two-sided<br>*Only common tests should be described solely by name; describe more complex techniques in the Methods section.*
☒	☐	A description of all covariates tested
☒	☐	A description of any assumptions or corrections, such as tests of normality and adjustment for multiple comparisons
☐	☒	A full description of the statistical parameters including central tendency (e.g. means) or other basic estimates (e.g. regression coefficient) AND variation (e.g. standard deviation) or associated estimates of uncertainty (e.g. confidence intervals)
☐	☒	For null hypothesis testing, the test statistic (e.g. *F*, *t*, *r*) with confidence intervals, effect sizes, degrees of freedom and *P* value noted<br>*Give P values as exact values whenever suitable.*
☒	☐	For Bayesian analysis, information on the choice of priors and Markov chain Monte Carlo settings
☒	☐	For hierarchical and complex designs, identification of the appropriate level for tests and full reporting of outcomes
☒	☐	Estimates of effect sizes (e.g. Cohen's *d*, Pearson's *r*), indicating how they were calculated

Our web collection on statistics for biologists contains articles on many of the points above.

Software and code

Policy information about availability of computer code

Data collection	Confocal images were obtained with a Nikon C2 microscope using the NIS Elements software. Images for western blot were taken using the Odyssey IR imaging system (LI-COR). ImageJ software (National Institute of Health) was used to quantify both microscopy and western blot images. qPCR results were obtained using Bio-Rad CFX 96. Adobe Photoshop 2022 and Illustrator 2022 software packages were used to assemble images into figures. Primers for qPCR were designed using Primer 3 online software. Virus replicon results were obtained using GLOMAXTM 96 microplate luminometer (Promega). For histology samples, images were acquired using AxioScan.Z1 The Transmission electron microscopy samples were examined with a JOEL JEM-1400Flash electron microscope and the images were recorded with a CCD camera (Morada G3, Emsis).
Data analysis	Statistical analyses were performed using the GraphPad Prism 9.0 or Microsoft Excel software. Unpaired two-tailed Student's t-test, one-way ANOVA, two-way ANOVA were used to assess statistical significance. Data sets were considered significantly different if the p value was less than 0.01.

For manuscripts utilizing custom algorithms or software that are central to the research but not yet described in published literature, software must be made available to editors and reviewers. We strongly encourage code deposition in a community repository (e.g. GitHub). See the Nature Portfolio guidelines for submitting code & software for further information.

Data

Policy information about availability of data

All manuscripts must include a data availability statement. This statement should provide the following information, where applicable:

- Accession codes, unique identifiers, or web links for publicly available datasets
- A description of any restrictions on data availability
- For clinical datasets or third party data, please ensure that the statement adheres to our policy

Materials generated in this study will be made available upon reasonable request and may require a material transfer agreement.

Research involving human participants, their data, or biological material

Policy information about studies with human participants or human data. See also policy information about sex, gender (identity/presentation), and sexual orientation and race, ethnicity and racism.

Reporting on sex and gender	N/A
Reporting on race, ethnicity, or other socially relevant groupings	N/A
Population characteristics	N/A
Recruitment	N/A
Ethics oversight	N/A

Note that full information on the approval of the study protocol must also be provided in the manuscript.

Field-specific reporting

Please select the one below that is the best fit for your research. If you are not sure, read the appropriate sections before making your selection.

☒ Life sciences ☐ Behavioural & social sciences ☐ Ecological, evolutionary & environmental sciences

For a reference copy of the document with all sections, see nature.com/documents/nr-reporting-summary-flat.pdf

Life sciences study design

All studies must disclose on these points even when the disclosure is negative.

Sample size	The sample sizes were chosen based on standard protocols in the field which allow for statistical determination of changes between given samples, and were indicated in the figure legends.(Song, GY., et al. Nat Commun 14, 6832 (2023); Goellner, S., et al. Nat Commun 14, 7344 (2023))
Data exclusions	No data were excluded from this manuscript.
Replication	All cell culture experiments were repeated multiple independent times. In vivo experiments were performed with independent repeat experiments.
Randomization	Mice used in the study were allocated randomly. In in vitro study using cultured cells, same number of cells were used for the experiments. The experiments were well-controlled.
Blinding	As the experimental groups were conducted in parallel with same procedures and data were quantitative, the investigators were not blinded. The data collection and analysis were performed carefully by at least two individual investigators and were blinded for all the experiments.

Reporting for specific materials, systems and methods

We require information from authors about some types of materials, experimental systems and methods used in many studies. Here, indicate whether each material, system or method listed is relevant to your study. If you are not sure if a list item applies to your research, read the appropriate section before selecting a response.

Materials & experimental systems

n/a	Involved in the study	
☐	☒	Antibodies
☐	☒	Eukaryotic cell lines
☒	☐	Palaeontology and archaeology
☐	☒	Animals and other organisms
☒	☐	Clinical data
☒	☐	Dual use research of concern
☒	☐	Plants

Methods

n/a	Involved in the study	
☒	☐	ChIP-seq
☐	☒	Flow cytometry
☒	☐	MRI-based neuroimaging

Antibodies

Antibodies used

Primary antibodies used include:
anti-SUN1 (Proteintech 24568-1-AP, 1:1000 for WB).
anti-SUN2 (Proteintech 27556-1-AP, 1:1000 for WB, 1:500 for IF).
anti-rabbit ZIKV envelope (GeneTex GTX133314, 1:1000 for WB, 1:500 for IF, 1:200 for IP, 1:2000 for IHC).
anti-mouse ZIKV envelope (GeneTex GTX634155, 1:1000 for WB, 1:500 for IF, 1:500 for flow cytometry).
anti-ZIKV NS1 (GeneTex GTX133307, 1:4000 for WB, 1:1000 for IF).
anti-ZIKV NS3 (GeneTex GTX133320, 1:1000 for WB).
anti-ZIKV NS4A (GeneTex GTX133704, 1:1000 for WB).
anti-ZIKV NS5 (GeneTex GTX133312, 1:1000 for WB).
anti-DENV2 NS1, mouse monoclonal Ab (A gift from Trai-Ming Yeh and Yung-Chun Chuang at National Cheng Kung University, 1:5000 for WB).
anti-HA (MBL M180-3, 1:2000 for WB).
anti-Calnexin (Proteintech 10427-2-AP, 1:1000 for IF).
anti-Nesprin-1 (Abcam Ab192234, 1:1000 for WB).
anti-Nesprin-1 (HUABIO ET7107-28, 1:1000 for IF).
anti-mCherry (Proteintech 26765-1-AP, 1:1000 for WB).
anti-GAPDH (Proteintech 10494-1-AP, 1:5000 for WB).
anti-α-Tubulin (Ray Antibody Biotech RM2007, 1:5000 for WB, 1:500 for IF).
anti-Vimentin (Proteintech 60330-1-Ig, 1:1000 for WB, 1:500 for IF).
anti-β-actin (Sigma A1978, 1:5000 for WB, 1:300 for IP).

Secondary antibodies used include:
IRDye 800 CW-conjugated anti-rabbit IgG (LI COR 926-32211, 1:5000 for WB)
IRDye 680 CW-conjugated anti-mouse IgG (LI COR 926-68020, 1:5000 for WB)
HRP-conjugated anti-mouse IgG (CST 51275, 1:5000 for WB)
goat anti-rabbit IgG secondary antibody (Alexa Fluor 488) (Invitrogen A-11008, 1:1000 for IF)
goat anti-mouse IgG secondary antibody (Alexa Fluor 647) (Invitrogen A-32728, 1:500 for IF, 1:1000 for flow cytometry)

Validation

Vendor validation information is available online for the following antibodies:
Rabbit anti-SUN1, Proteintech, Cat# 24568-1-AP
Rabbit anti-SUN2, Proteintech , Cat# 27556-1-AP
Rabbit anti-ZIKV envelope, GeneTex, Cat# GTX133314
Mouse anti-ZIKV envelope, GeneTex, Cat# GTX634155
Rabbit anti-ZIKV NS1, GeneTex, Cat# GTX133307
Rabbit anti-ZIKV NS3, GeneTex, Cat# GTX133320
Rabbit anti-ZIKV NS4A, GeneTex, Cat# GTX133704
Rabbit anti-ZIKV NS5, GeneTex, Cat# GTX133312
Mouse anti-HA, MBL, Cat# M180-3
Rabbit anti-Calnexin, Proteintech, Cat# 10427-2-AP
Rabbit anti-Nesprin-1, Abcam, Cat# Ab192234
Rabbit anti-Nesprin-1, HUABIO, Cat# ET7107-28
Rabbit anti-mCherry, Proteintech, Cat# 26765-1-AP
Rabbit anti-GAPDH, Proteintech, Cat# 10494-1-AP
Mouse anti-α-Tubulin, Ray Antibody Biotech, Cat# RM2007
Mouse anti-Vimentin, Proteintech, Cat# 60330-1-Ig
Mouse anti-β-actin, Sigma, Cat# A1978

Validation of mouse anti-DENV2 NS1 monoclonal Ab was performed using viral infection assay, which also validation in our previous study Wang Y, et al. J Virol. 2019 Feb 5;93(4):e01306-18.

Eukaryotic cell lines

Policy information about cell lines and Sex and Gender in Research

Cell line source(s)	A549, HeLa, 293T, Vero, BHK-21, and C6/36 were obtained from ATCC. Huh7 cells were provided by Dr. Yi-Ping Li (Sun Yat-sen University, Guangzhou, China).
Authentication	None of cell lines used in the manuscript were authenticated by authors.
Mycoplasma contamination	All cell lines are routinely tested for mycoplasma to ensure no contamination.
Commonly misidentified lines (See ICLAC register)	No commonly misidentified cell lines were used.

Animals and other research organisms

Policy information about studies involving animals; ARRIVE guidelines recommended for reporting animal research, and Sex and Gender in Research

Laboratory animals	Six-week-old specific pathogen-free wild type and Sun2-/- C57B/L6 mice were were purchased from GemPharmatech Co., Ltd and housed at 22±2°C and 50–60% relative humidity in a specific pathogen-free facility maintained on a 12 hours light/dark cycle at the research animal facility of Sun Yat-sen University. All neonatal animals were cage-bred with the mouse mothers during the experiment and observed daily until the end of assay. All mice were euthanized by cervical dislocation after the appearance of severe clinical signs, including lethargy and paralysis.
Wild animals	No wild animals were used in this study.
Reporting on sex	The sex information has not been collected.
Field-collected samples	The study did not involve field-collected samples.
Ethics oversight	All the experiments were performed with the approval by the Sun Yat-sen University Institutional Animal Care and Use Committee (SYSU IACUC) (approval number is 2021-000473).

Note that full information on the approval of the study protocol must also be provided in the manuscript.

Flow Cytometry

Plots

Confirm that:

☒ The axis labels state the marker and fluorochrome used (e.g. CD4-FITC).

☒ The axis scales are clearly visible. Include numbers along axes only for bottom left plot of group (a 'group' is an analysis of identical markers).

☒ All plots are contour plots with outliers or pseudocolor plots.

☒ A numerical value for number of cells or percentage (with statistics) is provided.

Methodology

Sample preparation	Cells were infected with ZIKV at an MOI of 3. At 24 h p.i., cells were suspended in PBS and incubated with ZIKV E antibody (1:500, GTX634155, GeneTex), followed by incubation with goat anti-mouse IgG secondary antibody (Alexa Fluor 647) (1:1000, A-32728, Invitrogen). Then, labeled cells were examined by flow cytometry.
Instrument	Beckman Coulter, CytoFLEX S
Software	Beckman Coulter CytExpert (version 2.4.0.28) software.
Cell population abundance	The investigators did not sort cells.
Gating strategy	Gating was used to eliminate debris and multiple cells using forward and side scatter parameters (FSC-A/FSC-A). The viral E protein Ab bound cells were gated positive based on ASC-A/FSC-A. The staining with isotype control was used as the control of gating.

☐ Tick this box to confirm that a figure exemplifying the gating strategy is provided in the Supplementary Information.
